# The miRNA–immune axis in bladder cancer: systematic evidence for a new era of immunotherapy precision

**DOI:** 10.3389/fimmu.2025.1639334

**Published:** 2025-09-19

**Authors:** Daniel-Vasile Dulf, Gloria Ravegnini, Federico Manuel Giorgi, Anamaria Larisa Burnar, Francesca Gorini, Antonio De Leo, Harisa Luţichievici, Constantin-Lucian Opriţa, Cezar-Nicolae Todiruţ, Tudor-Eliade Ciuleanu, Camelia Alexandra Coadă

**Affiliations:** ^1^ 10th Department - Oncology, “Iuliu Haţieganu” University of Medicine and Pharmacy, Cluj-Napoca, Romania; ^2^ Clinical Municipal Hospital Cluj-Napoca, Cluj-Napoca, Romania; ^3^ Department of Pharmacy and Biotechnology (FABIT), University of Bologna, Bologna, Italy; ^4^ Clinical Pharmacology Unit, IRCCS Azienda Ospedaliero-Universitaria di Bologna, Bologna, Italy; ^5^ Institute of Oncology “Prof. Dr. Ion Chiricuţă”, Cluj-Napoca, Romania; ^6^ Solid Tumor Molecular Pathology Laboratory, IRCCS Azienda Ospedaliero-Universitaria di Bologna, Bologna, Italy; ^7^ Department of Medical and Surgical Sciences (DIMEC), University of Bologna, Bologna, Italy; ^8^ Faculty of Medicine, University of Medicine and Pharmacy “Iuliu Haţieganu”, Cluj-Napoca, Romania; ^9^ Department of Morpho-functional Sciences, University of Medicine and Pharmacy “Iuliu Haţieganu”, Cluj-Napoca, Romania

**Keywords:** immune checkpoint inhibitor, immunotherapy, tumor immune infiltration, PD-L1, prognosis, urothelial carcinoma, personalized therapy, microRNA

## Abstract

**Introduction:**

Bladder cancer (BC) is a complex disease with patients showing widely variable responses to treatment. While immunotherapy has recently emerged as a promising alternative to the standard platinum-based chemotherapy, especially for platinum-resistant tumors, clinicians still lack reliable biomarkers to predict which patients will truly benefit from immunotherapy.

**Aim:**

This systematic review aimed to explore whether miRNAs could help decode the immune landscape of BC and serve as predictive biomarkers for immunotherapy response.

**Methods:**

A total of 3,272 articles were systematically screened on medical databases and narrowed down to 37 studies that examined the relationship between miRNAs, immune cell infiltration, and patient outcomes in BC. To further strengthen and validate our findings, we analyzed large-scale genomic data from The Cancer Genome Atlas (TCGA-BLCA).

**Results:**

A total of 104 different miRNAs appeared to shape the BC immune microenvironment. Some studies also linked miRNA expression with clinical outcomes such as BCG therapy response and prognosis, while others dissected the molecular pathways. Further analyses established miR-155, miR-142, and miR-146b as key factors for CD4^+^ memory T-cell and M1 macrophage infiltration. Notably, 49 miRNAs showed stage-specific expression differences in TCGA data, with 25 of them also significantly associated with progression-free interval or overall survival.

**Conclusion:**

miRNAs are emerging as powerful regulators of the immune microenvironment of BC. However, despite growing evidence, to date, no studies have directly explored miRNA profiles in driving immunotherapeutic decisions. Our findings highlight the need for prospective studies to translate these molecular insights into personalized treatment strategies.

## Graphical Abstract

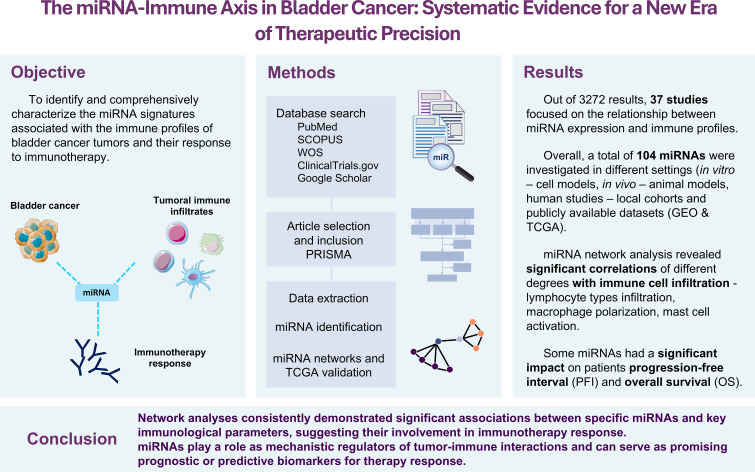

## Introduction

Bladder cancer remains one of the most prevalent malignancies of the urinary tract, with urothelial carcinoma accounting for over 90% of cases (1). Approximately 25% of patients are diagnosed with muscle-invasive bladder cancer (MIBC), a form associated with a worse prognosis (2). For these patients, the current standard treatment is represented by cisplatin-based neoadjuvant chemotherapy followed by cystectomy (2). Unfortunately, many patients experience disease progression or do not tolerate the treatment due to limited renal function, underscoring the urgent need for more effective therapeutic strategies (3). Recently, immunotherapy, such as immune checkpoint inhibitors (ICIs), has emerged as an alternative treatment for bladder cancer (in both neoadjuvant and adjuvant settings), with clinical trials evaluating the efficacy either alone or in combination with chemotherapy (4, 5). Despite the growing body of literature on this topic, no validated biomarkers are currently available in the clinical setting to identify bladder cancer patients who are most likely to benefit from immunotherapy, and a substantial proportion of patients exhibit limited or no durable response (4, 5).

MicroRNAs (miRNAs), a class of small non-coding RNAs that regulate gene expression at the post-transcriptional level, are well known for their modulating role in cancer biology (6). In bladder cancer, miRNAs have been implicated in key oncogenic processes, including proliferation, invasion, and epithelial-to-mesenchymal transition (7). Moreover, an increasing body of research is showing that miRNAs also play a role in regulating components of the tumoral microenvironment (TME), such as immune cell infiltration, expression of immune checkpoints, regulation of PD - 1/PD-L1 and CTLA - 4, and antigen presentation (2). These regulatory functions make miRNAs potential candidates as biomarkers for immune responsiveness as well as therapeutic agents.

The interplay between miRNAs and tumor immunity in bladder cancer remains largely incompletely understood. To date, a comprehensive characterization of miRNA-mediated immune regulation in the process of resistance to ICIs in bladder cancer is lacking.

In this study, we conducted a systematic review of available literature investigating the role of miRNAs in modulating the immune microenvironment within bladder cancer, as well as its response to immunotherapy, with the aim of synthesizing the current state-of-the-art knowledge on this topic. Furthermore, we comprehensively analyzed the relationship between the identified miRNAs, bladder cancer TME, and clinical profiles, to further strengthen the available level of evidence as well as to unravel novel, yet unexplored relationships that may lay the groundwork for precision immuno-oncology guided by miRNAs.

## Materials and methods

### Database search and study identification

A systematic literature search was performed to identify relevant studies investigating the relationship between bladder cancer, tumoral immune infiltration, immunotherapy, and miRNA. The search strategy employed a combination of targeted keywords, including “bladder cancer,” “urothelial carcinoma,” “immunotherapy,” “checkpoint inhibitor,” “immune response,” “immune evasion,” “immune infiltration,” “miR,” “microRNA,” “miRNA,” and “non-coding RNA.”

The databases used for the identification of relevant studies were PubMed, SCOPUS, Web of Science, ClinicalTrials.gov, and Google Scholar. Literature retrieval from Google Scholar was done via the Publish or Perish software (8), which facilitates structured bibliographic data extraction. The last updated search was conducted in March 2025.

### Study screening and inclusion criteria

The inclusion criteria were peer-reviewed original research articles published in English that addressed the role of miRNAs in the context of immunotherapy and/or immune mechanisms in bladder cancer. We considered studies including patients and animal models, as well as those conducted on cell lines. Case reports, editorials, and book chapters were excluded. The results from all databases were imported into Rayyan (9). Duplicates were removed, and titles and abstracts were screened manually by three authors for relevance. Full texts of eligible studies were downloaded and read in detail. Disagreements were resolved by discussion with a fourth researcher. Relevant data from each study were extracted and summarized in tables.

### TCGA data retrieval and analysis

Expression data (mRNA and miRNA) and clinical data for muscle-invasive urothelial carcinoma (pT2 or above) were obtained from The Cancer Genome Atlas (TCGA-BLCA) project using the *FirebrowseR* R package, via the Broad Institute’s Firehose pipeline. Only primary tumor samples with complete expression and clinical annotation were included in the subsequent analyses (10). Corresponding expression and clinical annotation files were integrated based on TCGA patient barcodes. Clinical data for survival analysis were obtained from the TCGA Pan-Cancer Clinical Data Resource (TCGA-CDR) (11).

### Tumoral immune cell populations and miRNA expression analysis

Immune cell infiltration estimates were obtained from the Tumor Immune Estimation Resource (TIMER) (7), a computational framework that deconvolutes the composition of the tumor immune microenvironment using bulk RNA-sequencing data. TIMER provides robust quantification of six key immune cell populations: B cells, CD4^+^ T cells, CD8^+^ T cells, neutrophils, macrophages, and dendritic cells. To ensure a comprehensive evaluation of immune infiltration, we utilized multiple established deconvolution algorithms that estimate immune cell abundance from the transcriptome data. The R packages *igraph* and *ggraph* were used for the construction of the networks between TME populations and miRNAs (12, 13).

### Statistical analysis

All statistical analyses were conducted in R version 4.4.3 (2025 - 02–28 ucrt) - “Trophy Case” (14). For correlation analyses, the Spearman rank correlation test was used. To enhance the strength of the findings and reduce the likelihood of false-positive results due to weak associations between variables of interest, a minimum correlation coefficient threshold of 0.3 was set to ensure a moderate to strong association. This cutoff was used to prioritize miRNAs with a potentially higher influence on the TME. All *p*-values were adjusted for multiple comparisons using the Benjamini–Hochberg procedure to control the false discovery rate (15).

For clinical analyses, one-way ANOVA tests were performed to compare miRNA expression levels between patient groups. To evaluate the prognostic impact of miRNAs, patients were stratified into high- and low-expression groups based on the median expression level of each miRNA. Progression-free interval (PFI) and overall survival (OS) were assessed using Kaplan–Meier survival curves and compared with the log-rank tests. Cox proportional hazards regression models were used to estimate the degree and significance of the association between miRNA expression and patient outcomes. A *p*-value of 0.05 was set for statistical significance.

## Results

**Figure 1:**
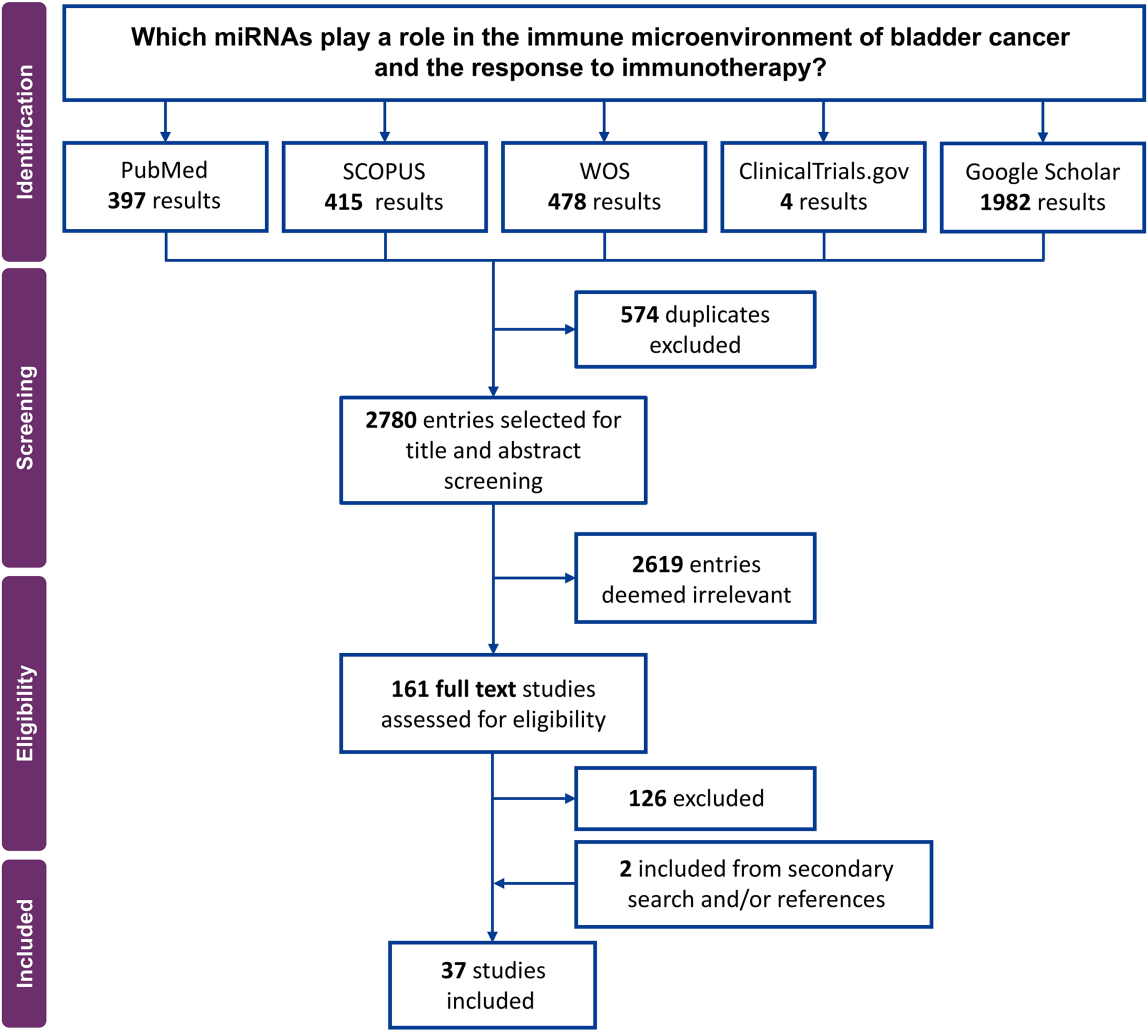
PRISMA chart showing the selection process of the articles included in this systematic review.

A total of 3,272 records were identified through the initial search. After removing 574 duplicates, 2,780 records remained for title and abstract screening. Following this step, 161 articles were deemed potentially eligible and retrieved for full-text review. Of these, 37 studies met the inclusion criteria and were included in the final analysis (**Figure 1**). Overall, the studies investigated a total of 104 different miRNAs (**Table 1**) using diverse cohorts and model systems. Several studies investigated the association between the expression levels of specific miRNAs and clinical patient characteristics such as OS and response to immunotherapy. Other studies focused on elucidating the molecular mechanisms by which these miRNAs regulate the TME, providing insights into their immunoregulatory functions and potential therapeutic relevance (**Table 1**, **Supplementary Table 1**).

**Table 1:** Summary of the studies exploring the miRNA–immune profiles in bladder cancer.

Author, year	Studied miRNAs	Effect on immune profile/major study outcomes
Ahanidi, 2022 (17)	miR-100-5p, miR-138-5p	miR-100-5p and miR-138-5p: strong inverse correlation with TERT, PD-L1, and PD-L2 expression, but not PD1.
Awadalla, 2022 (18)	miR-21, miR-31, let-7a, miR-199a	miR-31 and let-7a were lower, while miR-199a and miR-21 were upregulated in BCG non-responders; miR-21, STAG2, and NCOR1 were independent predictors of disease progression and BCG non-responders; lower miR-31 and ARID1A were independent predictors of disease recurrence and progression.
Bitting, 2024 (19)	miR-20, miR-125, miR-223, miR-21, miR-118	Lower levels of miR-20, miR-21, and miR-181 were associated with higher levels of CD4 INFg T lymphocytes; responders had lower levels of miR-20, miR-21, and miR-181.
Boubaker, 2020 (20)	let-7c, let-7g	No association with the neutrophil-to-lymphocyte ratio.
Cai, 2025 (21)	miR-490-5p, miR-204-3p	TNF signaling; Th17 cell differentiation. miR-490-5p was higher in the T1 stage; low expression of miR-490-3p and miR-204-3p was found in recurrent tumors.
Fan, 2022 (22)	miR-383	High CDH1 expression was negatively correlated with immune cell infiltration (pDCs, Tregs, T cells, macrophages, neutrophils, DCs, NK cells); EPCAM was positively correlated with CDH1 (regulated by miR-383).
Fu, 2021 (23)	miR-29c-3p, miR-374b-5p	Significant correlations between TTLL7, DSC2, ELN, and resting mast cells and hsa-miR-29c-3p and M0 macrophages.
Huang, 2023 (24)	miR-628-5p	miR-628-5p expression was significantly correlated with immune infiltration.
Huyan, 2022 (25)	miR-221-5p, miR-186-5p	Dysfunction and increased apoptosis of NK cells; miR-221-5p and miR-186-5p (exosomes) affect NK cell function by interfering with the stability of DAP10, CD96, and perforin mRNA.
Jiang, 2021 (26)	miR-1231-5p, miR-92b-3p	miR-1231-5p and miR-92b-3p inhibit PTEN expression and activate PI3K/AKT-STAT3/6 pathway to induce BMDM M2 differentiation to the immunosuppressive phenotype.
Jiang, 2020 (27)	miR-29c-3p, miR-20a-5p	miR-29c-3p negative correlation with neutrophils and follicular helper T cells; miR-20a-5p positive correlation with follicular helper T cells. ELN was negatively correlated with Th infiltration; DSC2 was positively correlated with neutrophil infiltration.
Jiang, 2020 (28)	miR-142, miR-223, miR-7702, miR-4772, miR-155, miR-150, miR-187, miR-429, miR-200a, miR-551b, miR-200b	miR-142, miR-223, miR-7702, miR-4772, miR-155, and miR-150 were the highest in the high-infiltration group; miR-187, miR-429, miR-200a, miR-551b, and miR-200b were lowest in the high-infiltrating group.
Kuo, 2024 (29)	miR-383-5p	High SUSD2 was associated with macrophage infiltration and M2 polarization; diminished IL - 2 signaling activation of CD8^+^ T cells and reduced their antitumor efficacy.
Li, 2024 (30)	miR-4786	Singlec-15 inhibited both IL - 2 and TNF-α; favored immune evasion; miR-4786 antagonizes BACH1-IT2 immune-suppressive effects.
Liu, 2022 (31)	miR-200a-3p	Low miR-200a-3p increased PD-L1 expression, consequently impacting NK cell killing efficacy, resulting in immunosuppression.
Liu, 2024 (31)	miR-107	BDNF positively correlated with M2 macrophages (CD206^+^) and inversely correlated with M1 macrophages (CD86^+^), which indicates that BDNF might drive an immunosuppressive TME by modulating M2 macrophage polarization.
Lu, 2021 (32)	miR-3666	miR-3666 overexpression inhibits susceptibility to NK cells.
Ke, 2021 (32)	miR-324, miR-191, miR-25, miR-93	Associated with cellular immune response (activation and chemotaxis of immune cells, such as neutrophil chemotaxis, monocyte chemotaxis, lymphocyte chemotaxis, and cellular response to interferon-gamma and interleukin-1); cell adhesion.
Lyu, 2022 (33)	miR-34a-5p	Positive correlations with MAP1A occurred for genes in B cells (CD19, CD79A), CD8^+^ T cells (CD8A, CD8B), CD4^+^ T cells (CD4), M1 macrophages (NOS2, PTGS2), M2 macrophages (CD163, VSIG4, MS4A4A), neutrophils (ITGAM, CCR7), and DCs (HLA-DPB1, HLA-DQB1, HLA-DAR, HLA-DPA1, CD1C, NRP1, ITGAX).
Martínez, 2017 (34)	miR-21	Polarization of M2-like macrophages.
Mei, 2024 (35)	miR-25, miR-548an	Neutrophils were more evident in the high-risk groups; regulatory T cells were increased in the low-risk group; immune cell functions (except type II IFN response) were more significant in the high-risk group.
Rao, 2022 (36)	miR-101-3p, miR-124-3p, miR-409-5p, miR-515-5p, miR-506-3p, miR-653-5p, miR-1252-5p	DLGAP5 had a positive correlation with Th2 cells and a negative correlation with NK CD56^+^.
Shi, 2024 (37)	miR-320a, miR-301b-3p	Promoted the production of immunosuppressive cytokines from PMN-MDSCs; BC-derived exosomes significantly inhibited the production of IFN-γ and the proliferation function of CD8^+^ T cells.
Song, 2022 (38)	miR-124-3p, miR-26b-5p, miR-192-5p	Enriched in pathways of immune response and immune cells, including the B-cell receptor signaling pathway, NK cell-mediated cytotoxicity, and T-cell receptor signaling pathway.
Stempor, 2021 (39)	miR-30d, miR-4778, miR-1306, miR-4756, miR-1287, miR-96, miR-3200, miR-187, miR-93, miR-423, miR-219a-1, miR-98, miR-182, miR-1307, miR-744, miR-301b, miR-940, miR-151a, miR-3193, miR-183, miR-191, miR-141, miR-7706, miR-200c, miR-429, miR-200b, miR-200a, miR-155, miR-146b, miR-142, miR-146a, miR-29a, miR-150, miR-4772, miR-5586, miR-223, miR-7702, miR-199b, miR-100, miR-199a-2, miR-511, miR-125b-1, miR-125b-2, miR-199a-1, miR-221, miR-21	27 microRNAs had opposite expression profiles to genes involved in immune response, and 24 microRNAs had similar expression profiles.
Wang, 2023 (40)	miR-125a-5p	PRPF19 had a positive correlation with mast cells and a negative correlation with stroma score, T-cell CD4, and T-cell CD8. PRPF19 exhibited predominant distribution in CD4 Tconv, CD8T, and NK cells (association with invading immune cells).
Wu, 2022 (41)	miR-590	High miR-590 was correlated with suppressed CD8^+^ T-cell infiltration and positively correlated with Treg infiltration. MED10 is a biomarker of local recurrence.
Xiong, 2022 (42)	miR-27a-3p	FASN inversely correlates with CD274. FASN was associated with the immune microenvironment, immune cell infiltration, and immune function in BC, and its CNV affected infiltration by CD4^+^ T cells, neutrophils, and dendritic cells. Patients with low FASN levels responded better to anti-PD-1 and CTLA4 treatments.
Yang, 2022 (43)	miR-141-3p, miR-200a-3p	CD8^+^ T-cell exhaustion leading to BC progression. miR-141-3p inhibition promoted proliferation and invasion.
Ying, 2025 (44)	miR-184	Promotes tumor growth *in vivo* through TIL-induced immunosuppression; miR-184 promotes T-cell exhaustion; CXCL10 inhibits tumors by promoting CD8^+^ T-cell infiltration.
Yuan, 2024 (45)	miR-17-5p	High expression of miR-17-5p was negatively correlated with NK cell infiltration, enhanced cell proliferation, migration, and invasion; increased miR-17-5p expression was linked to aggressive BC pathology; miR-17-5p promotes tumorigenesis *in vivo*.
Zhang, 2019 (46)	miR-30a	Increased miR-30a level was strongly associated with fewer CD133^+^; TAM promoted metastatic potential by EMT through the increase of miR-30a.
Zhang, 2021 (47)	let-7c, miR-99a, miR-125b-2, miR-200c	The TME score high group had significantly higher proportions of activated DC, monocytes, follicular helper T cells, and regulatory T cells; the TME score high group had a good prognosis and a good response to ICIs.
Zhang, 2024 (48)	miR-125b-2-3p, miR-145-3p, miR-409-3p, miR-625-3p	CD8^+^ T cells and CD4^+^ T cells: negative correlation with risk score, Treg cells, CD4^+^ memory T cells, mast cells, and M0 macrophages; positive correlation with risk score. let-7c-5p: positive correlation with resting mast cells and naive B cells; negative correlation with mast cell activation. miR-125b-2-3p: negative correlation with mast cell activation. miR-145-3p: positive correlation with naive B cells and resting mast cells; negative correlation with activated mast cells, activated memory CD4^+^ T cells, and CD8^+^ T cells. miR-409-3p: positive correlation with M0 macrophages, resting mast cells, and neutrophils; negative correlation with activated memory CD4^+^ T cells and CD8^+^ T cells. miR-548a-5p: negative correlation with plasma cells. miR-625-3p: positive correlation with follicular helper T cells, CD8^+^ T cells, and activated memory CD4^+^ T cells; negative correlation with Tregs, resting memory CD4^+^ T cells, neutrophils, monocytes, resting mast cells, and activated mast cells. miR-5682: negative correlation with neutrophils. All four miRNAs promoted bladder cell migration, invasion, and proliferation.
Zhao, 2021 (49)	miR-30c; miR-135a; miR-27a	Downregulation of miRNA promotes Cbl-b expression and deactivates T cells *in vitro*; Cbl-b overexpression contributes to tumoral immune escape.
Zheng, 2023 (50)	miR-328-3p	Upregulation of miR-328-3p decreases PD-L1.
Zhu, 2019 (51)	miR-145	ATG7 overexpression elevates PD-L1; PD-L1 was a critical downstream mediator for ATG7 overexpression, inducing stem-like properties, invasion, and anchorage-independent growth.

BC, bladder cancer; TME, tumor microenvironment; EMT, epithelial-to-mesenchymal transition; ICI, immune checkpoint inhibitor; Treg, regulatory T cells; NK, natural killer cells; TIL, tumor-infiltrating lymphocytes; Tconv, conventional T cells; Th, helper T cells; DC, dendritic cells.

Of the 104 studies in the literature, 20 have been classified by the Cancer miRNA Census (16) as oncomiRNAs (miR-155, miR-182, miR-183, miR-20a, miR-21, miR-25, miR-93, miR-96, and miR-17) and tumor suppressor miRNAs (miR-100, miR-138, miR-145, miR-204, miR-99a, let-7c, miR-101, miR-125a, miR-29c, miR-30a, and miR-30c) (**Supplementary Table 2**). Furthermore, these miRNAs have been demonstrated to be involved not only in immune evasion but also in other cancer hallmarks such as the p53 pathway, angiogenesis, apoptosis, epithelial-to-mesenchymal transition (EMT), and various signaling pathways such as KRAS and PI3K-AKT-mTOR (**Supplementary Table 2**).

### miRNA expression profiles associated with tumoral immune infiltration in the TCGA-bladder cancer cohort

**Figure 2:**
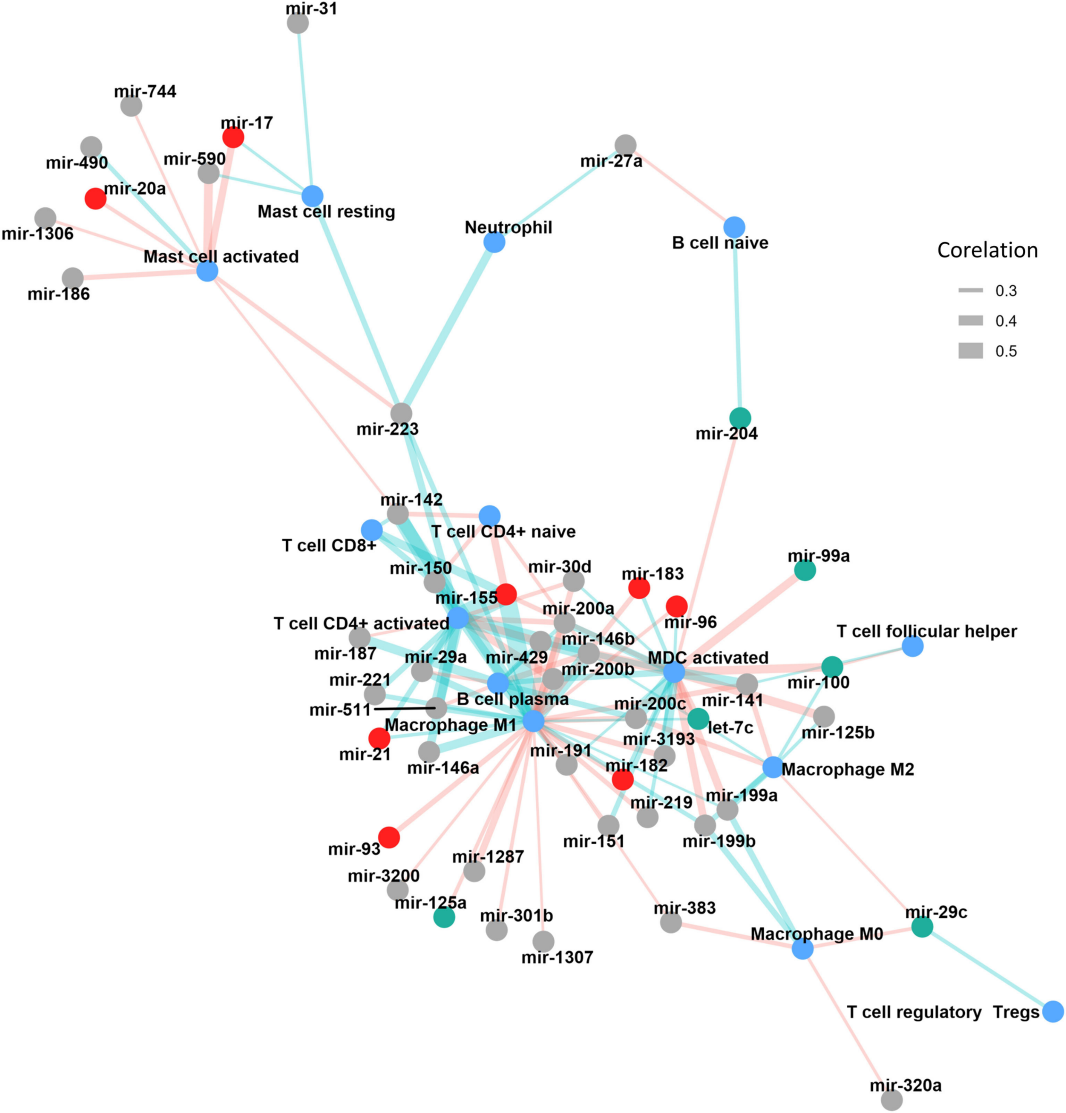
Correlation network illustrating the associations between miRNA expression and immune cell infiltration in TCGA-BLCA tumors. miRNAs are represented as nodes, with red indicating oncomiRs and green indicating tumor suppressor miRNAs. Immune cell populations are shown in blue. The thickness of the connecting edges reflects the absolute strength of the correlation, while the edge color denotes direction, light red for negative and light green for positive correlations. MDC, myeloid dendritic cells.

We identified a robust set of positive correlations between miRNA expression and immune cell infiltration in bladder cancer, highlighting potential immunoregulatory roles for these miRNAs. Among the most significant results, miR-155 had the strongest associations, showing high positive correlations with CD4^+^ T cells (*r* = 0.568) and M1 macrophages (*r* = 0.537). Similarly, miR-142, miR-146b, and miR-511 were strongly associated with CD4^+^ memory-activated T cells (*r* = 0.483, *r* = 0.477, and *r* = 0.452, respectively). miR-146b and miR-142 also significantly correlated with M1 macrophage infiltration (*r* = 0.453 and *r* = 0.441, respectively) (**Figure 2**, **Supplementary Table 3**).

Additional miRNAs, including members of the miR-200 family (miR-200c, miR-200a, miR-200b, and miR-429), demonstrated moderate correlations with activated myeloid dendritic cells (*r* ranging from 0.338 to 0.42), suggesting a role in dendritic cell-mediated immune responses. Other miRNAs such as miR-223, miR-29a, miR-150, and miR-146a showed moderate associations with various immune subsets, including neutrophils, T cells, and macrophages (**Figure 2**, **Supplementary Table 3**).

In contrast, a substantial number of miRNAs demonstrated moderate to strong negative correlations with immune cell subsets. Namely, miR-146b, miR-125b, and miR-100 exhibited strong inverse correlations with activated myeloid dendritic cells (correlation coefficients ranging from −0.39 to −0.37), while miR-200b, miR-200a, and miR-429 were negatively associated with M1 macrophages and activated CD4^+^ memory T cells. miR-590 and miR-17 also showed consistent negative correlations with activated mast cells. Interestingly, several miRNAs such as miR-200c, miR-141, miR-155, and miR-142 were negatively correlated with multiple immune cell types, highlighting their possible involvement in broader immune regulatory networks within the TME. This multiple regulation was also seen when analyzing how many different miRNAs correlated with each immune population (**Figure 2**, **Table 2**). M1 macrophages were the most frequently targeted immune cells, with 10 miRNAs being positively correlated with their degree of infiltration in the tumors and 13 miRNAs showing a negative correlation. CD4^+^ T cells were also correlated with multiple miRNAs (10 positively and 4 negatively correlated) (**Figure 2**, **Table 2**).

**Table 2:** Number of miRNAs with a significant correlation (*r* ≥ |0.3|, adjusted *p*-value < 0.05) with tumoral immune infiltrates in the TCGA-BLCA cohort.

Tumor immune cell populations	Number of miRNAs with:	
	Positive correlation	Negative correlation
Activated myeloid dendritic cells	8	8
M0 macrophages	2	1
M1 macrophages	10	13
M2 macrophages	2	2
Activated memory CD4^+^ T cells	10	4
Naive CD4^+^ T cells	0	3
CD8^+^ T cells	2	0
Plasma B cells	5	1
Naive B cells	1	0
Activated mast cells	1	4
Resting mast cells	1	0
Neutrophils	1	0

### Impact of miRNA levels on the TCGA bladder cancer patients’ outcomes

**Figure 3:**
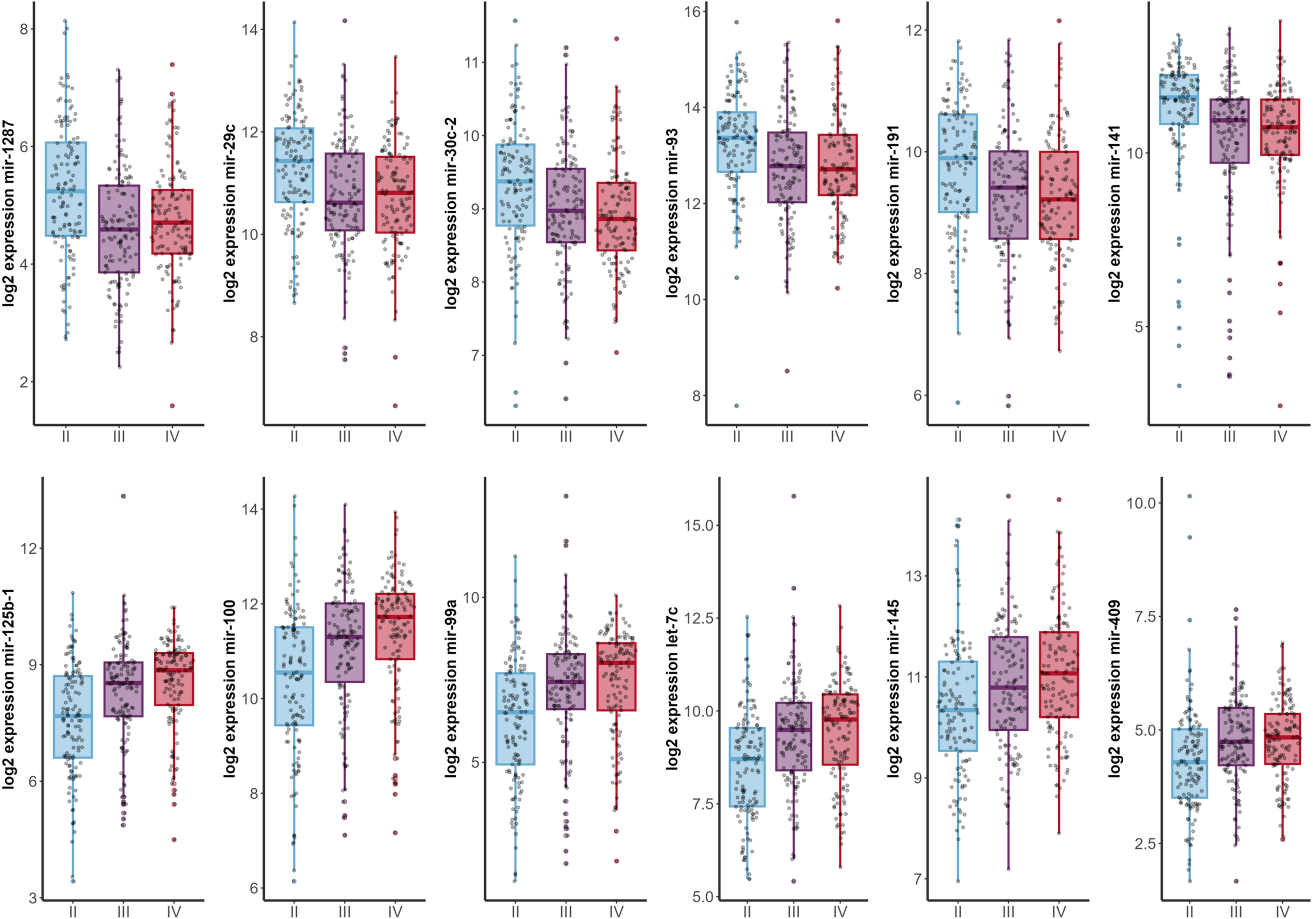
Expression profiles of highly significant miRNAs (*p* < 0.001) across different tumor stages (x-axis). Stage I patients were excluded from the analysis due to insufficient sample size (*n*=2).

Analysis of miRNA expression across different tumor stages yielded a total of 49 miRNAs with significant differences (**Supplementary Table 4**). Of these, 13 were also significantly associated with patients’ prognosis (**Supplementary Tables 5**, **6**). Notably, six miRNAs (miR-1278, miR-29c, miR-30c, miR-93, miR-191, and miR-141) exhibited a decreasing expression trend, with the highest levels observed in stage II and the lowest in stage IV. Conversely, six other miRNAs (miR-125b, miR-100, miR-99a, let-7c, miR-145, and miR-409) showed increasing expression levels from stage II to the more advanced stages (**Figure 3**, **Supplementary Table 4**).

**Figure 4:**
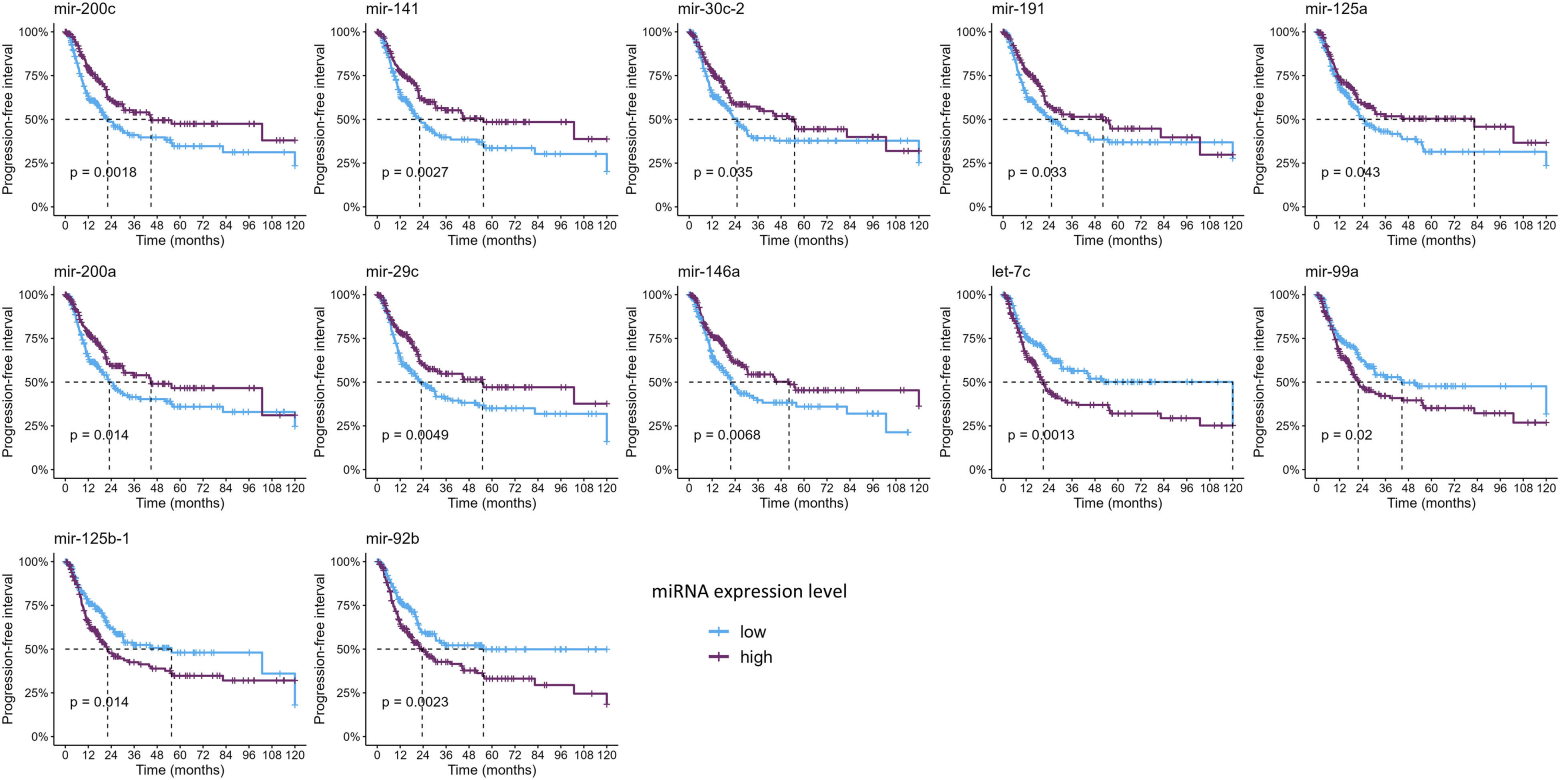
Kaplan–Meier curves showing the miRNAs significantly associated with progression-free interval (*p*-values were computed with the log-rank test).

**Figure 5:**
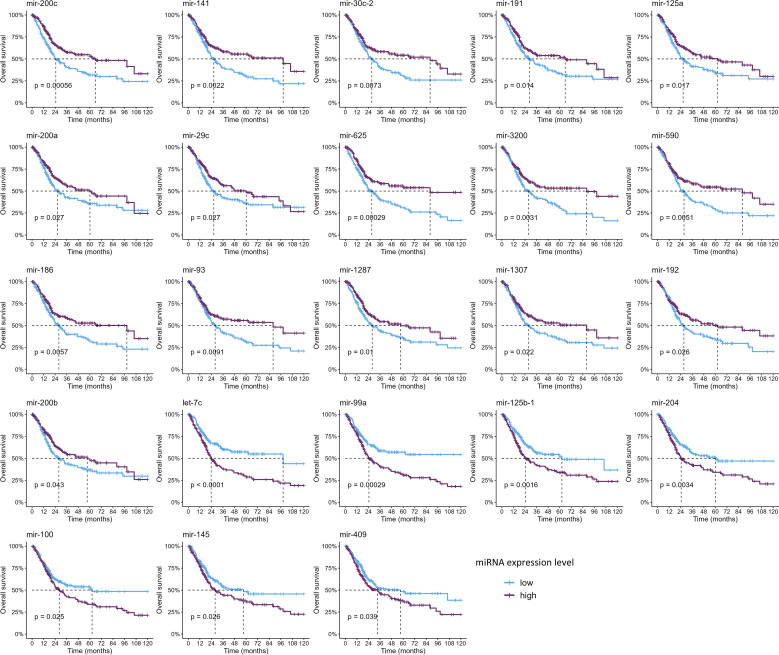
Kaplan–Meier curves showing the miRNAs significantly associated with overall survival (*p*-values were computed with the log-rank test).

Since only nine studies evaluated the impact of miRNAs on OS and only one on PFS, we sought to analyze these data on the TCGA-BLCA cohort to further evaluate the clinical relevance of these miRNAs (**Figures 4**, **5**, **Supplementary Tables 5**, **6**). Out of all miRNAs analyzed, 12 were significantly associated with PFI, and 23 were significantly associated with OS. Specifically, elevated expression levels of miR-200c, miR-141, miR-30c, miR-191, miR-125a, miR-200a, and miR-29c were associated with improved PFI and OS. Additionally, higher levels of miR-625, miR-3200, miR-590, miR-186, miR-93, miR-1287, miR-1307, miR-192, and miR-200b were significantly associated with better OS. Conversely, increased expression of let-7c, miR-99a, and miR-125b correlated with poorer PFI and OS. Higher levels of miR-204, miR-100, miR-145, and miR-409 were linked to worse OS. Notably, elevated miR-92b expression was associated with a shorter PFI, although its association with OS did not reach statistical significance (**Table 3**, **Supplementary Tables 5**, **6**).

**Table 3:** Cox proportional hazards for PFI and OS of patients from the TCGA-BLCA project.

miRNAs	PFI		OS	
	HR 95%CI	*P*-value	HR 95%CI	*P*-value
mir-29c	0.65 (0.48, 0.88)	0.005	0.71 (0.53, 0.96)	0.028
mir-30c	0.72 (0.54, 0.98)	0.036	0.67 (0.49, 0.9)	0.008
mir-93			0.67 (0.5, 0.91)	0.010
mir-125a	0.73 (0.54, 0.99)	0.044	0.7 (0.52, 0.94)	0.017
mir-141	0.63 (0.47, 0.85)	0.003	0.63 (0.46, 0.85)	0.002
mir-146a	0.66 (0.49, 0.89)	0.007		
mir-186			0.66 (0.49, 0.89)	0.006
mir-191	0.72 (0.53, 0.98)	0.034	0.69 (0.51, 0.93)	0.015
mir-192			0.71 (0.53, 0.96)	0.027
mir-200a	0.68 (0.51, 0.93)	0.015	0.71 (0.53, 0.96)	0.028
mir-200b			0.73 (0.54, 0.99)	0.044
mir-200c	0.62 (0.46, 0.84)	0.002	0.59 (0.44, 0.8)	0.001
mir-590			0.65 (0.49, 0.88)	0.005
mir-625			0.57 (0.42, 0.78)	<0.001
mir-1287			0.68 (0.5, 0.91)	0.011
mir-1307			0.71 (0.52, 0.95)	0.023
mir-3200			0.64 (0.47, 0.86)	0.003
let-7c	1.64 (1.21, 2.22)	0.001	1.88 (1.38, 2.55)	<0.001
mir-92b	1.6 (1.18, 2.18)	0.003		
mir-99a	1.43 (1.06, 1.94)	0.020	1.75 (1.29, 2.39)	<0.001
mir-100			1.41 (1.04, 1.9)	0.026
mir-125b	1.46 (1.08, 1.97)	0.015	1.62 (1.2, 2.2)	0.002
mir-145			1.4 (1.04, 1.9)	0.027
mir-204			1.56 (1.16, 2.11)	0.004
mir-409			1.37 (1.01, 1.85)	0.040

For each miRNA, patients with higher levels were compared to those with lower levels (i.e., the reference group).

HR, hazard ratio; CI, confidence interval.

## Discussion

### Summary of main findings

The results of this systematic review and comprehensive analysis highlight a complex network of interactions between specific miRNAs and components of the bladder cancer TME, which is yet to be fully explored and understood. These findings, completed by the bioinformatic analyses aimed at filling the literature gaps, support their potential as modulators of immune infiltration in bladder cancer. Although different perspectives have been explored, there are still insufficient data to definitively demonstrate the value of miRNAs in the selection of patients undergoing immunotherapy.

While numerous studies have investigated the role of individual miRNAs in bladder cancer immunity, their focus has often been fragmented, with different studies examining different miRNAs and distinct immune cell populations, very few on checkpoint molecules, and with limited clinical endpoints. For instance, miR-100-5p and miR-138-5p were linked to PD-L1/PD-L2 regulation (17), while others, such as miR-21 and miR-31, were associated with BCG response, disease progression, and recurrence (18), but no validation studies to confirm these results were found. Additional studies explored the interplay between select miRNAs and specific immune subsets, including CD4^+^ IFN-γ^+^ T cells, NK cells, macrophages, and regulatory T cells (19, 22, 26, 28). However, these investigations frequently lacked integration across cell types, mechanisms, and clinical impact. Moreover, only a few studies examined the prognostic significance of immune-related miRNAs systematically and comparatively.

Our study addresses these gaps by providing a comprehensive synthesis of the roles of the 104 miRNAs implicated in shaping the immune microenvironment of bladder cancer. By integrating findings from the published experimental models and validating them with large-scale data (TCGA-BLCA), we connected the dots between immune modulation and clinical outcomes such as PFI and OS. This integrative approach summarizes the current state-of-the-art on the immunoregulatory role of miRNAs in bladder cancer and offers a more unified view for understanding their potential impact on immunotherapy response. Moreover, this work lays the ground for their future use as prognostic and potentially predictive biomarkers.

### miRNAs as modulators of the immune microenvironment and response

The differential expression of specific miRNAs and their association with immune cell infiltration patterns highlight the potential of miRNAs as both biomarkers and functional regulators of immune dynamics (52). These observations align with the broader framework of tumor immunoediting, a dynamic process encompassing the phases of elimination, equilibrium, and escape, through which cancer cells continuously evolve to evade immune surveillance (53).

**Figure 6:**
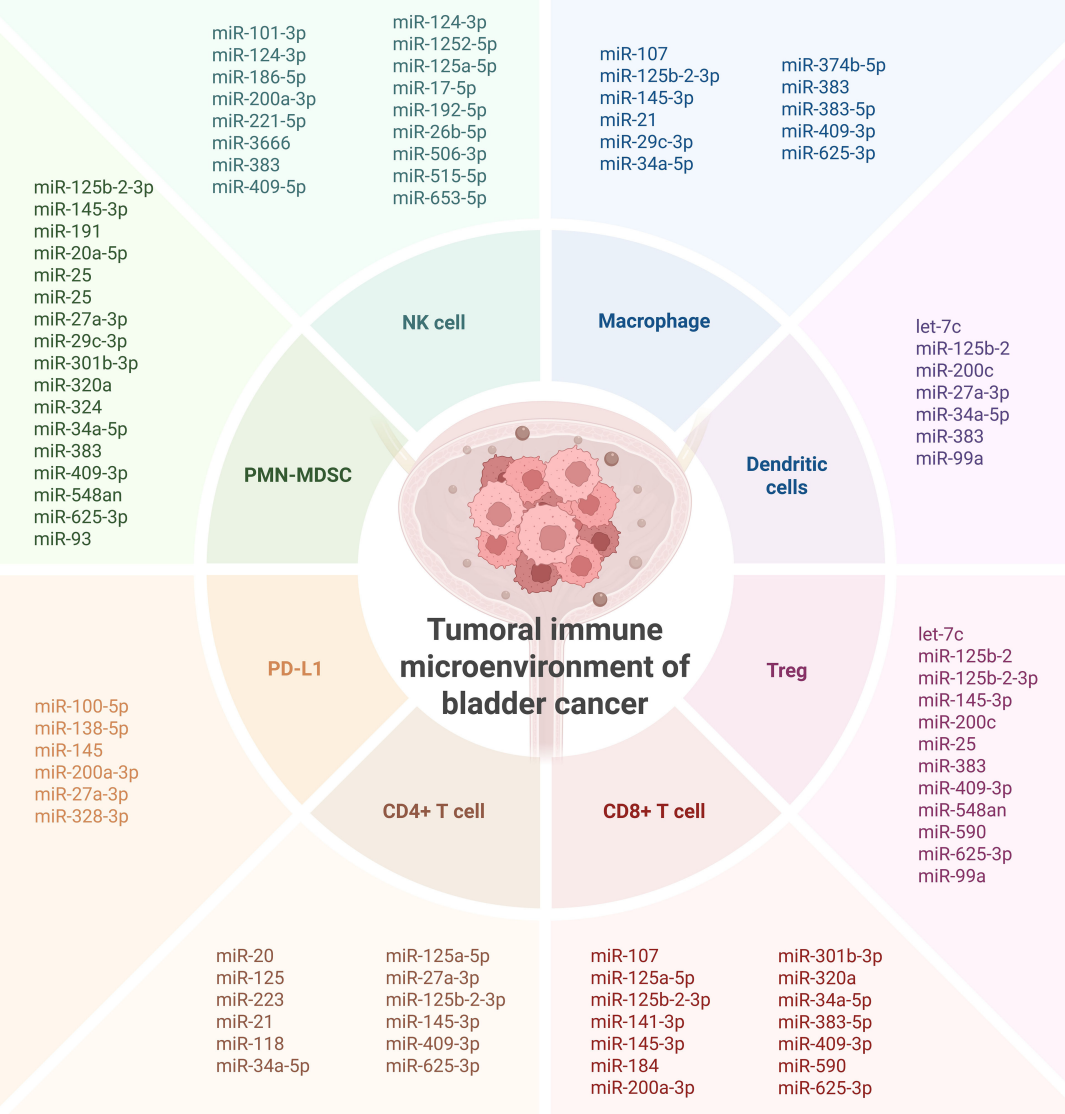
Overall presentation of the investigated miRNAs involved in shaping the immune microenvironment of bladder cancer. Created in BioRender. Coada, C. (2025) https://BioRender.com/dknj33y.

In the escape phase, immune evasion becomes dominant, and tumors acquire multiple mechanisms to suppress antitumor immunity. Many of the miRNAs identified in this review are likely active participants in this phase. For instance, expression of miR-21 and miR-141-3p correlates with diminished cytotoxic immune cell infiltration (e.g., CD8^+^ T cells, NK cells), suggesting a role in establishing immune exclusion or immune suppression (18, 19, 34, 39, 43). Similarly, downregulation of miRNAs such as miR-29c-3p and miR-186-5p, which have been linked to immune activation, may further support the development of an immunosuppressive TME (23, 25) (**Figure 6**).

The heterogeneity of miRNA expression patterns observed across patient cohorts also reflects the broader variability in immune infiltration and response observed in bladder tumors. The bladder cancer TME frequently harbors immunosuppressive components, including tumor-associated macrophages (TAMs), regulatory T cells (Tregs), and myeloid-derived suppressor cells (MDSCs), all of which can be recruited and sustained via miRNA-regulated signaling pathways (54). Moreover, the enrichment of immune checkpoint ligands such as PD-L1 and PD-L2 in miRNA-associated gene networks suggests a role for miRNAs in modulating immune checkpoint pathways, which are central to current immunotherapeutic strategies. High expression of PD-L1, often driven by oncogenic or hypoxia-induced signaling, is further modulated by miRNAs such as miR-138-5p and miR-100-5p (54). Loss or suppression of these miRNAs may contribute to the upregulation of immune inhibitory signals, allowing tumor cells to escape T-cell-mediated cytotoxicity (**Figure 6**).

The immunosuppressive TME interferes with normal immune responses and, thus, can reduce the efficacy of immune checkpoint blockade, which has been recently approved for bladder cancer treatment (55). Our review suggests that miRNA expression profiles could potentially serve as predictive biomarkers for immunotherapy response, as evidenced, for example, by their association with BCG failure (e.g., miR-21, miR-31) (18) (**Figure 6**).

Importantly, the functional links between miRNAs and immune regulation open up new opportunities for therapeutic intervention. Targeting specific miRNAs could complement existing immunotherapies by reversing immunosuppressive programming or restoring antitumor immunity. For example, miRNA mimics or antagomirs could be employed to modulate key pathways associated with T-cell recruitment, macrophage polarization, or checkpoint expression, thus enhancing the depth and durability of immune responses (54).

### Implications for current medical practice and future directions

With the highly positive results obtained by immunotherapy in other cancers (54) and the approval of novel therapies for bladder cancer, research focusing on identifying the most promising candidates who would benefit from it is highly needed. Significant progress has been made in the use of miRNAs as biomarkers in bladder cancer, including the validation of a panel capable of diagnosing and stratifying patients (7). However, whether these panels or similar ones can also identify responders to treatment remains an open question. Of note, several of the miRNAs (miR-221-3p, miR-93-5p, miR-191-5p, miR-200c-3p, miR-192-5p, miR-21-5p) included in this panel have already been investigated for their roles in tumor immunity, suggesting that the development of miRNA-based tools to predict immunotherapy response is a feasible and promising avenue.

Emerging evidence highlights the critical role of miRNAs (often transported via tumor-derived exosomes) in preparing the pre-metastatic niche, particularly by modulating immune landscapes at distant sites (56). These miRNA-loaded exosomes act as messengers that condition the future metastatic environment, promoting immune evasion, suppressing cytotoxic T-cell activity, and recruiting immunosuppressive cells such as regulatory T cells, M2 macrophages, and myeloid-derived suppressor cells (56, 57). For example, certain miRNAs can inhibit antigen presentation by downregulating MHC class I molecules or modulate cytokine production to create a tolerogenic or “immune-silent” microenvironment that favors metastatic colonization. This immune reprogramming not only facilitates tumor cell seeding and outgrowth at distant sites but also contributes to resistance against immunotherapies such as immune checkpoint blockade (56, 57). By converting immunologically “hot” environments into “cold” niches, these miRNAs help shield disseminated tumor cells from T-cell-mediated clearance. Understanding and targeting these miRNA-mediated pathways offer a novel opportunity for disrupting the metastatic cascade and enhancing immunotherapeutic efficacy through restoration of immune surveillance in both primary and secondary tumor sites (53, 58).

miRNAs play multifaceted roles in modulating immune infiltration in bladder cancer and are increasingly recognized as promising biomarkers. Although increasing evidence shows their potential clinical relevance in immune-oncology, no studies to date have directly assessed miRNA profiles in patients receiving immunotherapy. Prospective studies and robust clinical validation are urgently needed to assess their utility as reliable biomarkers of immunotherapy response in bladder cancer patients.

### Study limitations

While this systematic review and integrated TCGA analysis provide a comprehensive overview of the miRNA–immune axis in bladder cancer, several limitations should be mentioned to contextualize our findings and guide future research. The primary studies included in this systematic review had considerable heterogeneity. This was evident in the diversity of miRNAs investigated, the variety of experimental models used (ranging from *in vitro* cell lines to animal models and human tumor tissues), the different immune cell populations assessed, and the clinical or biological endpoints reported. Such heterogeneity, while reflective of the exploratory nature of much of the research on this topic, prevented any meaningful meta-analysis and therefore requires caution when concluding from this combined literature.

Moreover, our TCGA-BLCA data analysis, while robust, has some limitations as well. The use of bulk RNA-sequencing data for immune cell deconvolution provides estimates of immune cell abundance but cannot fully show the spatial organization or functional state of these cells within the tumor microenvironment. The associations identified between miRNA expression, immune infiltration, and clinical outcomes are also correlational and do not imply causality. While these findings provide valuable hypotheses, they require experimental validation on other cohorts.

Despite the promising associations identified, a significant translational gap remains. The gap from identifying significant miRNA expression patterns or *in vitro*/*in vivo* mechanistic links to clinically validated biomarkers predictive of immunotherapy response is quite wide. The current body of evidence, as synthesized here, highlights the need for prospective, well-designed clinical trials to validate the utility of these miRNAs in guiding therapeutic decisions in bladder cancer.

## Conclusion

This systematic review shows the emerging and multifaceted role of miRNAs in modulating the immune landscape of cancer.

Network analyses consistently demonstrated significant associations between specific miRNAs and key immunological parameters, including lymphocyte infiltration, macrophage polarization, and mast cell activation. Several miRNAs were linked to oncological endpoints, showing a strong impact on PFI and OS.

These findings underscore the potential of miRNAs as both mechanistic regulators of tumor–immune interactions and as promising prognostic and/or predictive biomarkers. However, further studies are required to evaluate their potential role as predictors for immunotherapy response.

## Data Availability

The original contributions presented in the study are included in the article/**Supplementary Material**. Further inquiries can be directed to the corresponding author.
